# Modelling the perspectives of distance education students towards online learning during COVID-19 pandemic

**DOI:** 10.1186/s40561-022-00193-y

**Published:** 2022-03-09

**Authors:** Moses Segbenya, Brandford Bervell, Vincent Mensah Minadzi, Beatrice Asante Somuah

**Affiliations:** 1 Department of Business Studies, College of Distance Education, University of Cape Coast, Cape Coast, Ghana; 2 Department of Mathematics, Science and ICT, College of Distance Education, University of Cape Coast, Cape Coast, Ghana; 3 Department of Education, College of Distance Education, University of Cape Coast, Cape Coast, Ghana; 4 Centre for Instructional Technology and Multimedia, Universiti Sains Malaysiat, Penang, Malaysia

**Keywords:** Online learning, Use, Prospects, Challenges, Online gadgets, Distance education

## Abstract

This study examined perspectives of distance education students on online learning based on their access gadgets (availability and functionality), online presentation or teaching methods, actual use of online learning coupled with its challenges. A quantitative approach and descriptive survey design were adopted for the study. A sample of 1061 was drawn from a study population of 44,134 distance learners across sixteen administrative regions and data collected using a questionnaire. Partial least squares structural equation modelling (PLS-SEM) was used to analyse the hypotheses of the study, and descriptive statistics were deployed to analyse students perceived challenges. The study found a statistically significant relationship between the availability and functionality of online gadgets, availability of online gadgets and online presentation methods, functionality and online presentation methods and functionality and online learning usage. Thus, these factors are necessary for successful online learning. Four main perceived challenges identified were unreliable power/electricity and internet connectivity, lack of collaboration and motivation during online learning. Therefore, it was recommended that steps are taken to resolve the perceived challenges while adopting the blended approach comprising face-to-face and online learning. The blended learning approach would enable students to adjust better and appreciate its usefulness in distance education.

**Supplementary Information:**

The online version contains supplementary material available at 10.1186/s40561-022-00193-y.

## Introduction

Higher educational institutions globally face the urgent need to provide diverse learning opportunities for students to learn. The urgency is occasioned by the emergence of the Covid-19 pandemic in the later part of 2019 (Liu et al., 2020; Aboagye et al., 2020). Educational institutions worldwide had to partially or wholly switch to online learning mode to keep teaching and learning ongoing (Adedoyin & Soykan, 2020; Tanveer, 2020; Mpungose, 2020). Online learning is a type of learning where learning experiences occur in synchronous or asynchronous environments using different resources such as mobile phones and laptops where there is internet availability (Dhawan, 2020). On his part, Al-Busaidi (2013) conceptualised online/e-learning as the delivery of learning using the internet and digital facilities. Ahmad (2012), on the other hand, said that online/e-learning is all about learning with the use of technologies, presumably computers and other modern-day tools. Students can be anywhere to learn and interact with instructors and colleague students (Singh & Thurman, 2019).

Online learning is relatively new within the Ghanaian context; however, the Covid-19 pandemic has required its urgent application across all educational levels. Distance education institutions in Ghana, including the University of Cape Coast, University of Ghana and University of Education, were not spared by the effect of the pandemic. Due to COVID-19 related restrictions on mass gatherings, students could not meet for the scheduled weekend’s face-to-face tutorial sessions due to the closure of educational institutions in the country. Several events that required personal contacts had to be shifted to the virtual platforms to curtail person to person contacts.

Though face-to-face contacts were restricted to curb the spread of the virus, online teaching and learning activities in all educational settings were, however, allowed to take place. Online learning should not pose a challenge to distance education students (who hitherto depended on only face-to-face interaction) since, by the nature of the programme, the greater part of the learning is done by the students anytime and anywhere on their own. Distance students meet over the weekends to interact with course facilitators to clarify concepts and principles students find difficult in the reading materials. So, online learning such as platform would naturally suit the characteristics of the distance learner. (Online learning in this study is an alternative to face-to-face learning in the distance education mode where teaching and learning is done through platforms including Zoom and Google meets, with the aid of internet and computers or android phones).

Effective teaching, either traditional face-to-face or online learning in higher education, is influenced by six factors exemplified by Kira and Saade (2006). These variables are the affect, a learner’s perception of the course, a perceived learning outcome, an attitude, an intrinsic motivation and an extrinsic motivation. This means that students’ attitude toward online learning is one of the most crucial considerations in determining their intention to adopt online learning in any context (Ja’ashan, 2020). Thus, the operationalisation of the intended purely online learning by distance educations in Ghana could be successful if students’ interest and by extension, affect, perception of the online course, perceived learning outcome, attitude, and motivation are considered by the management of these institutions before full implementation.

Meanwhile, online learning in developing economies is confronted with infrastructure-related challenges such as the availability and functionality of online tools, irregular electricity and internet facilities, and requisite skills to participate in online learning platforms (Yilmaz, 2016; Rapanta et al., 2020). Thus, the need for distance education institutions to switch to online learning as a result of the emergence of the Covid-19 pandemic will depend on the availability and functionality of online tools, type of online presentation methods and the usefulness of online learning to students (Dube, 2020; Elumalai et al., 2020). These factors may have the propensity to influence students’ online intention and acceptance of the intended purely online learning in Ghana. However, little has been empirically done in Ghana to seek distance students’ perspectives and readiness to accept the intended new learning mode (online learning) during the pandemic. Within this background, the current study was undertaken to examine students’ online intention towards online learning for distanRapantace learners due to the COVID-19. The study was guided by four hypotheses which were:

### H1

There is a statistically significant effect of the availability of online gadgets on the functionality of online gadgets.

### H2

There is a statistically significant effect of the availability of online gadgets on online teaching/presentation methods.

### H3

There is a statistically significant effect of the functionality of online gadgets on online teaching/presentation methods.

### H4

There is a statistically significant effect of the functionality of online gadgets on the actual use of online learning platforms.

Apart from the four hypotheses, the study also sought to examine perceived challenges among students towards online learning.

## Literature review

### Theoretical and empirical perspectives

This study was underpinned by the theory of reasoned action (TRA) developed by Ajzen and Fishbein (1980). The TRA is premised on the assumption that the immediate determinant of behaviour is an intention, or what is termed as behavioural intention (BI). The theory implies that several factors are considered before certain actions take place, specifically people’s attitudes and subjective decisions (Ajzen & Fishbein, 1980; Fishbein & Ajzen, 1975). Furthermore, this theory is based upon two basic assumptions: first, people are rational, and second, their social actions are under will control. The rational aspect indicates that human beings use the information available to them, and the model is based on the premise that social interactions are primarily guided by reasoning and behaviour (Ajzen, 1991). So, students’ attitude towards an online may be positive evaluation or negative evaluation of engaging in an online programme. Therefore, online learning intention among students could be influenced by their evaluation of the usefulness of online learning to improve academic work, availability and functionality of online tools, and the competency in engaging in online learning.

### Prospects of online learning

Concerns about the efficacy of online learning have been raised in relation to face-to-face learning mode (Panyajamorn et al., 2018; Nguyen, 2015). There is no doubt that online learning could offer wide-ranging opportunities for students to learn if they have the prerequisite skills, the device and positive attitudes towards it. For example, it provides flexibility (Smedley, 2010), interactivity (Leszczyński et al., 2018), and self-pacing (Amer, 2007). Within this context, a number of studies were carried out to address the concerns of stakeholders involved in higher education. Muthuprasad et al. (2020) investigated students’ perception and preference for online education in India during the COVID-19 pandemic. Their investigation indicated that the flexibility and convenience of online lessons are prospects that make online learning an attractive option since it enables students to work at their own pace.

Similarly, a study by Oluniyi (2012) found that flexibility was one of the prospects outlined by respondents. Another study by Arkorful and Abaidoo (2014) showed that online learning is good for slow learners and motivates students to learn as they can interact and exchange ideas with tutors or colleagues via the internet. They further found that online learning facilitates learning, implying that tutors can teach very well through online learning. Moreover, a study by Cantoni, Cellario, and Porta as cited in Coman et al. (2020) revealed benefits that students can derive from online learning, including saving time and money since it does not involve travelling to and from the learning centres. Additionally, Olukayode (2015) conducted a study in Nigeria and found that prospects of online learning were in the area of promoting distance education, extending the frontiers of knowledge, eradicating illiteracy, and making education more effective.

### Availability and functionality of online gadgets and online intention

The availability of online devices or gadgets to the implementation of an online programme is well researched. For instance, Alzahrani and O’toole (2017) study indicated that students who had online devices had significantly more positive attitudes towards accepting online education programmes. This means that students could access any online platforms to engage in learning if there is a need. Their finding was consistent with other studies by Al Otaibi (2012); Al Mahmud (2011) which revealed a positive effect of online device accessibility and/or experience on student attitudes towards it. When students have the necessary internet devices and can use them for online learning, they would accept to engage in online learning programmes (Afolayan, 2015; Leszczyński et al., 2018).

Students’ intention to accept online learning programmes is influenced by their perceived usefulness to improve their academic work. It has been established that students’ perceived usefulness has been an influential determinant of students’ intention to accept online programmes (Guritno & Siringoringo, 2013). The implication, therefore, is that if students believe that technology use would improve their academic performance, they would have positive attitudes towards the implementation of online programmes. A study conducted by Vululleh (2018) to validate the extended technology acceptance model by pre-tertiary and tertiary institutions in Liberia revealed that students’ intention to accept and use online learning in Liberia was significantly affected by their perceived usefulness.

### Challenges confronting effective online learning

Online learning comes with challenges irrespective of the status of development (Sarvestani et al., 2019). This means that even the most developed countries also have their own share of the challenge. Challenges with implementing successful online learning programmes can be put into infrastructure, institutional and personal. Mac-Ikemenjima (2005) explained the infrastructure as including computer hardware and software, and bandwidth access, lack of skilled workforce to manage available systems and inadequate training facilities for ICT education at the tertiary level. Course challenges related to content, design and delivery, characteristics of the individual student or the teacher, technological challenges, contextual challenges related to organisational, cultural and societal (Anderson & Grönlund, 2009). Oye et al. (2011) added that there is inadequate or non-availability of internet access and limited bandwidth in some tertiary institutions. In developing countries like Ghana, the possible challenges to be encountered in introducing an online programme are poor technical infrastructure, financial restrictions, lack of computer literacy, internet connectivity, energy-related problem, and limited expertise (Anene et al., 2014; Abaidoo & Arkorful 2014).

Additionally, a study by Nambiar (2020) to examine the impact of online learning during COVID-19 revealed that students indicated distraction of online classes as some of the challenges they faced. Nambiar (2020) found that when students do not have a conducive learning environment for an online class, it affects their effective participation, which may later impact negatively on their performance. The study of Nambiar (2020) further indicated that being at home makes online classes burdensome as they cannot manage both housework and college work simultaneously. Combining online classes with other social realities definitely could put pressure on students. A study by Khan et al. (2021) to investigate students’ perception and challenges online revealed that lack of motivation to take online classes is one of their major problems.

### Online intention among students as compared to traditional f2f

Nambiar (2020) found that 87.1% of the students preferred the traditional classroom face-to-face teaching method more than the online teaching mode, while 12.9% preferred online classes. This was confirmed in a study by Abbasi et al. (2020), who highlighted that student are not prepared to accept e-learning. Their finding, however, was contradictory to the study by Ali et al. (2016), whose study indicated that e-learning was a better teaching tool and was preferred by students. Paechter and Maier (2010), in their study, found that Austrian students still preferred face-to-face learning for communication purposes and the preservation of interpersonal relations.

Inversely, researchers such as Ryan et al. (2016), González-Gómez et al. (2016), Southard et al. (2015) found in their meta-study that students in blended programmes achieved better results than students in traditional classrooms. This indicates that the fear of stakeholders about the efficacy of online learning is allayed. Moreover, Adams et al. (2018) also investigated students’ readiness for a blended learning model of instruction (both face-to-face sessions and online learning approaches) in a leading Malaysian higher education institution. The results of the study showed that students in this higher education institution were ready for online learning and had the required technological skills. This could be because students had the much-needed technological skills due to prior training on information technology and exposure to technological innovations, which made them tech-savvy.

**Fig. 1:**
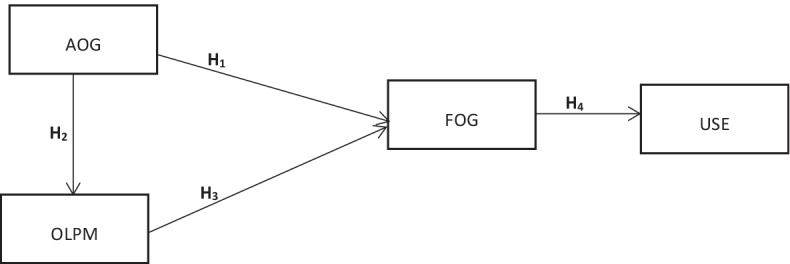
Conceptual framework highlighting the dependent and independent variables of the study. *AOG* Availability of Gadgets; *FOG* Functionality of gadgets; *OLPM* Online teaching/presentation methods; *USE* Use of online learning platform

The conceptual discussion in terms of the variables of interest to this study is portrayed in Fig. 1. The Figure also shows the interconnectedness of the hypotheses of the study.

## Methodology

The study adopted the quantitative approach and the descriptive survey design. A sample of 1061 was drawn from a study population of 44,134 distance learners of a college, across sixteen administrative regions. The sample represents 2.4% of the study population which was far higher as compared to the 381 recommended by Krejcie and Morgan’s (1970) and will therefore give a better representation (Segbenya & Ahiatrogah, 2018). Simple random and stratified sampling techniques were deployed to administer the research instrument. These sampling techniques were deployed to provide equal chances to all subjects in the population and for the purpose of representations from all levels of students on a distance education programme. Based on available sample frame (students list), a random number was generated and assigned to each student on the student list using excel software. This was followed by selecting respondents until the required number was obtained (simple random). Meanwhile, the required number of respondents was proportionately distributed among the various levels of students, type of programme and degree being pursued (stratified sampling) with the help of the sampling frame (students list).

The data collection instrument was a self-developed and self-distributed questionnaire. The questionnaire had two parts. The first part focused on the demographic characteristics of respondents, while the second part dealt with the five variables of the study that constituted the hypotheses as well as challenges. The questionnaire was measured on four points Likert Scale such as strongly agree, agreed, disagree and strongly disagree (Additional file 1). The reliability of the instrument was checked, and all the variables of the study scored a Cronbach alpha value above 0.70 thresholds suggesting that the instrument was good to be used. Data collection lasted for four months, starting in March and ending in June 2021. All ethical considerations, such as informed consent, privacy, freedom to opt-out despite starting the process and anonymity were discussed with respondents and agreed upon before data was collected. Data were analysed with PLS-Structural Equation Modelling to test the hypotheses, and descriptive statistics were used to analyse students’ perceived challenges.

**Fig. 2:**
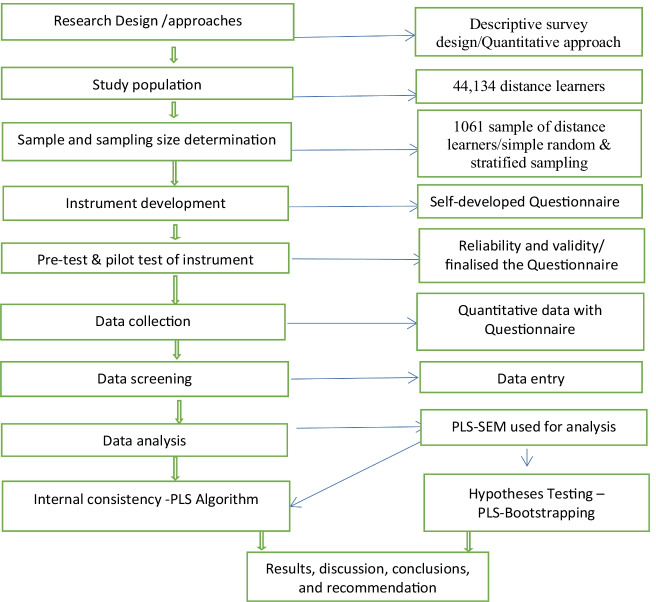
The methodological process highlighting the various stages of study

The methodological process of the study has been summarized in Fig. 2, comprising ten stages starting from the research design and approaches to the presentation of the findings, conclusion, and recommendation. Each of the first nine steps on the left side of the figure was equally linked to the specific activity taken as indicated on the right side of the figure. Meanwhile, at specific activity eight for stage eight (Data analysis), the instrument's internal consistency for measuring the model was checked with PLS-Algorithm and that of hypothesis testing for path system was also determined with PLS-Bootstrapping, after which results, discussion, conclusion and recommendation were captured.

## Analysis and findings

The first part of the results presents the demographic characteristics of respondents as presented in Table 1. It must be noted that the undergraduate programmes of the University selected for the study was divided into two parts- foundational level termed as Diploma and the Top-up level also termed as First Degree. The foundational level takes three years, and the top-up takes two years, making five years for the entire undergraduate programme. The results indicate that majority of the respondents were male students (52.0%), were in their fourth year (31.2%), pursuing education-related undergraduate programmes (69.7%). The majority of the respondents were also Diploma students (58.6%).

**Table 1:** Demographic characteristics of students. *Source*: Field survey (2021), N = 1061

Variables	Categories	Frequency	Percent
Category of students	Student	913	86.1
	Students National Executive	9	0.8
	Class Representative (Rep)	97	9.1
	Study Centre Students’ Rep	42	3.9
	Total	1061	100.0
Gender	Male	552	52.0
	Female	509	48.0
	Total	1061	100.0
Level	1st year	213	20.1
	2nd year	80	7.5
	3rd year	269	25.4
	4th year	331	31.2
	5th year	168	15.9
	Total	1061	100.0
Type of academic programmes	Education	740	69.7
	Business	252	23.8
	Maths and Science	69	6.5
	Total	1061	100.0
Category of Degree	Diploma	622	58.6
	Bachelor’s Degree	439	41.4
	Total	1061	100.0

**Fig. 3:**
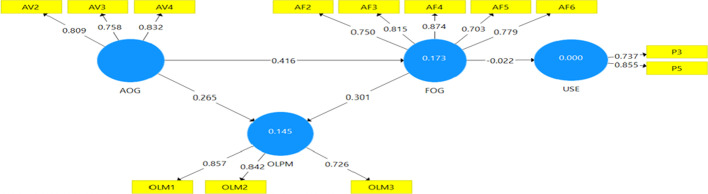
Confirmatory factor analysis. *Source*: Field survey (2021)

The second part of the results focused on the four hypotheses guiding the study. The hypotheses were tested with the use of PLS structural equation modelling. The initial assessment of the Partial Least Square Structural Equation algorithm was done with confirmatory factor analysis (CFA) and the results are presented in Fig. 3. From Fig. 3, only items that loaded 0.70 and above for their respective variables were used to enhance the strength of the model. Figure 3 also presents the loadings for each item, measuring each variable used for this study. The CFA in Fig. 3 clearly shows that all items loaded above the 0.70 minimum threshold.

### Internal consistency measure for the measurement model

Reliability check and convergent discriminant validity were checked to assess the internal consistency of the SEM model. The two main elements used to check the model’s reliability were Cronbach’s Alpha and composite reliability. Average variance Extracted [AVE] was also used to check the convergent discriminant validity of the PLS model. Henselaer et al. (2015) criterion of Cronbach alpha and composite reliability value of 0.70 and above and a value of 0.50 and above were used as thresholds, and the results can be seen in Table 2. The results in Table 2 show that the Cronbach alpha values obtained were between 0.723 and 0.849, except the variable “usage” that had a 0.439 Cronbach alpha. This means that the model had achieved reliability.

**Table 2:** Internal consistency measure for variables of the study. *Source*: Field survey (2021)

Items	Loadings	Cronbach’s alpha	rho_A	Composite reliability	Average variance extracted (AVE)
AF2	0.750	0.849	0.867	0.889	0.618
AF3	0.815				
AF4	0.874				
AF5	0.703				
AF6	0.778				
AV2	0.809	0.723	0.736	0.842	0.641
AV3	0.758				
AV4	0.832				
OLM1	0.857	0.736	0.748	0.851	0.657
OLM2	0.842				
OLM3	0.726				
Use3	0.819	0.439	0.447	0.778	0.638
Use5	0.781				

The “usage” variable was not deleted because, together with the rest of the variables of the study, they had composite reliability within the acceptable threshold of above 0.50. The composite reliability value obtained for all the four variables of the study were within 0.778–0.889, suggesting high reliability of constructs. The average variance estimate (AVE) was used to determine the convergent validity and the results can be seen in Table 2. The results show that AVE values obtained for all study variables were between 0.618 and 0.657 and were far above the 0.50 *minimum threshold,* suggesting convergent validity of the variables was attained for the study.

### Discriminant validity

How each construct found in the model differ from other constructs was determined by using the Heterotrait-Monotrait Ratio (HTMT) based on the suggested criterion of Henseler et al. (2015) cited in Bervell and Arkorful (2020). The criterion for recording zero (0) for diagonal loading on the same construct and less than 0.85 between different constructs. Table 3, therefore, presents the results for HTMT) and it is clear that all HTMT values were between 0.030 and 0.473 between constructs and zero for the same constructs. The results in Table 3 means that discriminant validity was achieved for this study.

**Table 3:** Heterotrait-monotrait ratio (HTMT). *Source*: Field survey (2021)

Variables	AOG	FOG	OLPM	USE
AOG	**0**			
FOG	0.473	**0**		
OLPM	0.354	0.439	**0**	
USE	0.030	0.036	0.043	**0**

The bolded zero (0) values mean that there is no discrimination of the same variable on itself

### Multicollinearity

Multicollinearity check carried for the constructs using Variance Inflated Factor (VIF) produced results shown in Table 4. The results suggest that all values obtained were *below 3.0,* implying that there was no multicollinearity threat.

**Table 4:** Multicollinearity (inner VIF values). *Source*: Field survey (2021)

Variables	AOG	FOG	OLPM	USE
AOG		**1.000**	**1.210**	
FOG			**1.210**	**1.000**
OLPM				
USE				

The bolded values in Table 4 are the VIF values that were all below the 3.0 threshold

### Path analysis

**Fig. 4:**
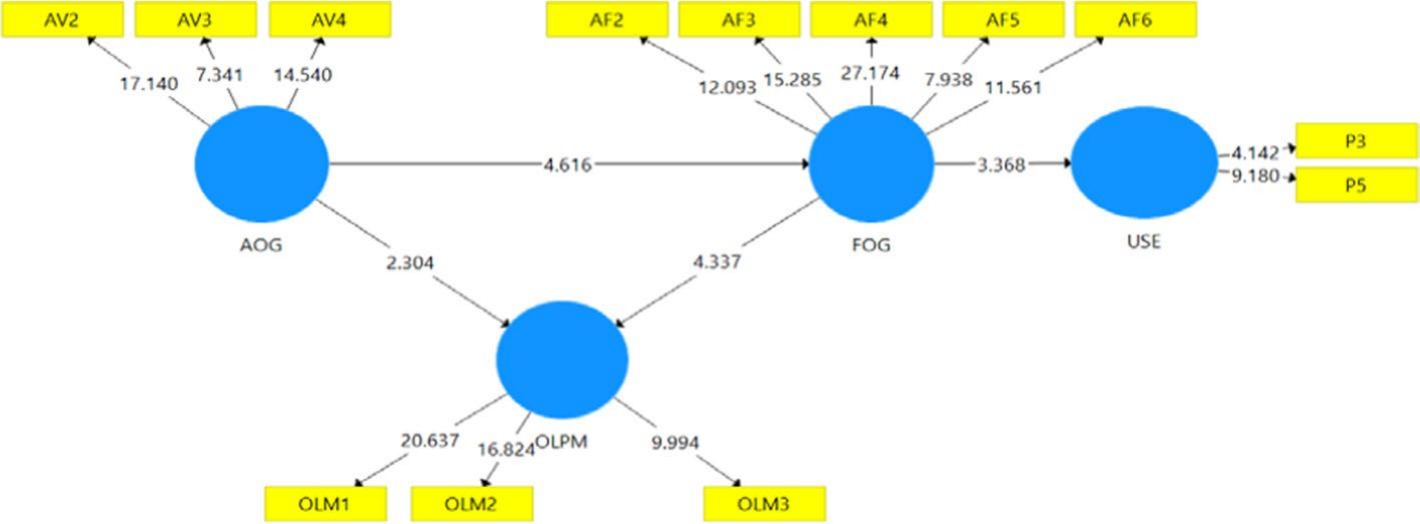
Results of Bootstrapping obtained for path analysis. *Source*: Field survey (2021)

The path significance of the model was determined, and the results are presented in Fig. 4. The graphical presentation in Fig. 4 agrees with Hair et al. (2017) suggestions of using a bootstrapping sequence of 5000 samples to assess a structural model. Thus, the PLS-bootstrapping results obtained from PLS-SEM in Fig. 4 helped in the path system to pave way for the actual path analysis for testing of the hypotheses guiding the study.

### Results of path analysis and testing of hypotheses

Results for the four hypotheses can be found in Table 5. The results from the tested hypotheses for the study are therefore presented in Table 5. The results revealed that Hypothesis 1, which connote that the availability of online gadgets has a significant relationship with the functionality of online gadgets (*β* = 0.416, *t* = 4.619, *p* = 0.000); Hypothesis 2, which states an availability of online gadgets has a significant relationship with the online presentation method (*β* = 0.140, *t* = 2.243, *p* = 0.025) were all supported.

**Table 5:** Path significance. *Source*: Field survey (2021)

Relationships	Beta coefficient	Sample mean (M)	Standard deviation (STDEV)	*T* statistics	*p* values	Confidence intervals		
						f^2^	2.5%	97.5%
AOG—> FOG	0.416	0.429	0.090	4.619	0.000**	0.210	0.234	0.597
AOG—> OLPM	0.140	0.141	0.062	2.243	0.025*	0.019	0.025	0.261
FOG—> OLPM	0.301	0.312	0.073	4.143	0.000**	0.087	0.171	0.462
FOG—> USE	0.022	0.022	0.006	3.566	0.000**	0.000	0.036	0.011

**p < 0.000, *p < 0.05 supported

Also, Hypotheses 3 which states that the functionality of online gadgets significantly relates to the online presentation method (*β* = 0.301, *t* = 4.143, *p* = 0.000), and hypothesis 4 that relates to the functionality of online tools significantly predicting online usefulness (*β* = 0.022, *t* = 3.566, *p* = 0.000), were all supported because there was a significant relationship between the two variables.

The effect size of the significance of the study variables was also checked, and the results for the effect size as indicated by f^2^ indicates that there were minor and moderate effects. Cohen (1988) cited in Bervell and Arkorful (2020) that effect sizes of 0.02, 0.15, and 0.35 above indicate small, medium, and large significant effects were used as a criterion. The results show that the effect size (*f*^2^) ranged between 0.019 and 0.210, respectively, for this study. This was further confirmed by the confidence intervals recorded in terms of lower and upper boundary values at a 95% confidence, indicating no false effect of the prediction of the significance obtained for variables of the study.

### Challenges associated with online learning

The study further sought to find out about students perceived challenges with online education, and the descriptive results can be seen in Table 6. It must be noted that for the results presented in Table 6, responses for ‘Agree’ was a combination of ‘strongly agree’ and ‘agree’ responses on the questionnaire. Similarly, the ‘Disagree’ responses in Table 6 is also an aggregate of ‘disagree’ and ‘strongly disagree’ responses captured on the questionnaire. The results indicate that respondents agreed with all the seven items used to measure the perceived challenges associated with online teaching. Notable among students’ challenges were that students might lose the opportunities to collaborate with other students if e-learning is introduced (77.3%), students might not be motivated during online teachings as compared to face-to-face teaching (76.3%), finding a quiet place to join online classes could be challenging for student (73.6%), unreliable power-electricity could disrupt online teaching and learning (73.2%).

**Table 6:** Challenges associated with online learning. *Source:* Field survey (2021)

Challenges	Disagree	Agree	Total
I might not be able to prepare well for e-learning	30.2	69.7	100
I might not be able to participate in e-learning effectively as compared to Face-to-face	33.5	66.5	100
I might lose the opportunities to collaborate with other students if e-learning introduces	22.7	77.3	100
Keeping students interested in the course content during e-learning lessons could be challenging for students	34	66	100
Making students feel included as a member of the class during online teaching might not be possible	36.7	63.2	100
Students might not be motivated during online teachings as compared to face-to-face teaching	23.7	76.3	100
Finding a quiet place to join online classes could be challenging for me	26.5	73.6	100
Combining online learning with family responsibilities, especially if I have to join online classes from home, will be challenging for me	34.5	65.6	100
Combining online teaching with work schedules, especially if I have to join online classes from the workplace, might be challenging for me	28.6	71.5	100
Online classes could reduce the availability of tutors to help students	32.6	67.5	100
I might perform poorly academically because of e-learning/online classes	40.6	59.4	100
I have not been trained for online learning	32.5	67.5	100
I do not have the prerequisite skills for online classes	36.3	63.8	1001
I might not get the requisite logistics for the online lessons (computer, laptop, tablets and Android phone)	38	62	100
Unreliable internet connectivity could disrupt teaching and learning whiles online	24.1	76	100
Unreliable power-electricity could disrupt online teaching and learning	26.9	73.2	100
Online teaching will not allow students to be able to discuss topics with their classmates	31.7	68.3	100

Other challenges were combining online teaching with work schedules, especially if one has to join online classes from the workplace, which might be challenging (71.5%), unreliable internet connectivity could disrupt teaching and learning while online (76%), were also crucial challenges or fears learners have indicated could serve as barriers to their participation in the intended purely online education.

## Discussion on findings

The study’s finding that the availability of online gadgets significantly influences the functionality of online gadgets suggests that online facilitation thrives on online gadgets. Thus, the role of online gadgets in ensuring successful online facilitation cannot be underestimated. These findings agree with Alzahrani and O’toole (2017) study, which indicated that students who had online devices had significantly more positive attitudes towards accepting online education programmes.

The relevance of online gadgets implies that individual students involved in distance education must take the necessary steps to acquire these essential online tools/gadgets such as Android phones, laptops, and desktop computers, among others, to participate in online facilitation. This might become a hurdle for learners since some distance learners could hardly pay their school fees without external support. Thus, the expenditure to procure online gadgets has become an essential additional cost to their education if they opt for it or are mandatorily required to join the intended purely online learning. This finding also corroborates the findings of a study by Al Otaibi (2012) that the inability to afford the cost of online gadgets could negatively influences participation in online education.

The finding for the second hypothesis of the study that the availability of online gadgets influences online presentation methods suggest that individual students’ ability to participate in any online facilitation methods largely hinges on online tools. There are several online presentation methods such as audio recorded lectures, video/voiceover PPT (PowerPoint Slides) and lived online lectures through the learning management system, zoom or google meet. Online presentation methods could generally be classified into synchronous and asynchronous online presentations methods. Irrespective of the method/s to be adopted, this study has found that the availability of online gadgets is key to students participating in either synchronous and asynchronous online education. Thus, the assertion by Al Mahmud (2011) that the availability of online gadgets significantly influences acceptance of online among students is upheld by this study.

The results also mean that the kind of online presentation method to be adopted for online facilitation should consider tools available to users of the online platform. Some online tools can best work with asynchronous online methods such as emails, discussion boards/forums, wikis, podcasting and e-portfolios, among others. Other online tools that can best work synchronously with live lectures include MOOC, zoom, google meet, google hangouts, among others. Thus, the ability of distance learners to participate in synchronous or asynchronous lectures will largely be dependent on the kind of online gadgets available or in possession of them.

The results of hypothesis three on the significant influence of functionality of online gadgets on online presentation methods further enhance the discourse on the subject. Choosing a particular online presentation method (either synchronously or asynchronously) is not only contingent upon the availability of online gadgets, but rather, the available gadgets must be in a good state or functioning state to perform the online activities that will be required. Meanwhile, the functionality of online gadgets is very dependent on the availability of consistent power sources- electricity and reliable internet connectivity (Anderson & Grönlund, 2009). Students can either depend on national or institutional internet connectivity provided. Alternatively, students can supplement the limited and unreliable national or institutional internet connectivity available by hotspot private internet connectivity on their mobile phones. This latter option comes with additional cost to distance learners who are already struggling to pay their school fees. The findings agree with that of Oye et al. (2011) that the cost of data and reliable internet connectivity influences the acceptance of online learning among students. A blended approach where both online and face-to-face tutorial sessions are used could be another alternative in the absence of reliable internet connectivity and electricity.

The significant relationship established between the functionality of online gadgets and the usefulness of online platforms to distance learners is very important. The results show that the functionality of online gadgets determines the usefulness that students will attach to online education on the distance mode. That is to say that learners cannot adduce the advantages of online facilitation without functioning online gadgets. There are several usefulness of online education to students, such as reducing the difficulties associated with travelling to the study centre for face-to-face sessions (Mesesan-Schmitz et al., 2020). Others are making learning occur anytime, anywhere and greatly improve knowledge retention and meet the learning needs of different categories of students (Mesesan-Schmitz et al., 2020). Meanwhile, distance learners can only take advantage of these wonderful outcomes of online learning when they have functioning online gadgets. The results, therefore, is in tandem with that of Alzahrani and O’toole (2017) that functioning online gadgets significantly influences online presentation methods.

The perceived challenges associated with the intended purely online education among students identified by this study can serve as barriers to implementing purely online education. The first of these challenges was that students might lose the opportunities to collaborate with other students. This means that students fear that gains made through collaborative learning on the face-to-face mode will not be available on the online teaching platforms. The second most severe challenge to implementing online facilitation from students’ perspective was the fear that students might not be motivated during online teachings as compared to face-to-face teaching.

Students' motivation is necessary for sustained interest in online facilitation amidst other challenges (Arkorful & Abaidoo, 2014). The motivation could be internal or external. The third severe challenge identified was unreliable internet connectivity that could disrupt teaching and learning whiles online. Another significant challenge to online education identified by this study was unreliable power-electricity that could disrupt online teaching and learning. The challenge of regular power or electricity seems very pervasive as compared to the earlier challenges identified. The functionality of all the online gadgets and to derive all the usefulness associated with online facilitation largely depend on the availability of a consistent and reliable source of power/ electricity.

## Theoretical implications

The study's findings have a theoretical implication based on the theory of reasoned action (TRA). The first implication is that students consider several factors to make academic decisions. Thus, for students to opt for online learning, key factors should be considered: the availability and functionality of online tools such as laptops, desktop computers, and tablets. Other factors include reliable internet facilities and electricity to determine whether to go ahead with the existing face-to-face tutorial sessions, use a blended approach (both face-to-face sessions and online learning approaches) or go ahead with purely online learning as envisaged.

The second theoretical implication of the study is based on the fact that distance learners are rational beings who make decisions and take actions based on available information to them. This means that vigorous and progressive orientation or education is needed to convince students about the usefulness of the intended purely online learning. Another dimension of the kind of information students need is training on how to use online gadgets to participate in online education. This requires time and consistent efforts to achieve the intended results with these activities.

## Implications for policy and practice

The implication of this study’s findings has several implications for management and the human resource managers for distance education institutions. Firstly, despite all the benefits of online education, students could not identify themselves with these benefits. This means that students are not very much convinced that the kind of online facilitation in Ghana could provide them with such benefits. It means that management and its human resource managers must consider how the intended purely online facilitation could provide such benefits to students. Secondly, the implication of the findings of the study suggests that management has a role to play in providing online gadgets for distance learners to participate in online education. Management could do this directly by adding the cost of these online gadgets to students’ fees or encouraging student to buy these gadgets prior to gaining admission or starting the academic work.

Another implication of the results for management is that students need the necessary skills to be able to participate in online facilitation. Thus, students need training on how to use online tools to participate in online teaching. The implication of all the challenges identified is that students were hesitant about the purely online education and were unwilling to participate until the challenges were dealt with. Management ability to collaborate with the key stakeholders to resolve the identified challenges is key for successfully implementing the intended purely online facilitation.

## Conclusion and recommendations

This study was carried out to examine the COVID-19 pandemic and intended purely online education for distance learners in Ghana. It can be concluded that the key factors to a successful implementation of the intended purely online education in Ghana will largely depend on the availability of online gadgets, the functionality of online gadgets, accessible online presentation methods, the usefulness of online education to students. Thus, the study found that there was a statistically significant relationship between the availability of online gadgets and functionality of online gadgets, availability of online gadgets and online presentation methods. Another relevant factor was the relationship between the functionality of online gadgets and online presentation methods and the functionality of online gadgets and online usage. Challenges identified that were responsible for the insignificant relationship were unreliable electricity and internet connectivity, fear of collaboration, and motivation for students during online lectures.

It is therefore recommended that the management of distance education institutions in Ghana should assist distance learners in procuring online tools to be able to participate in online facilitation. Management could do this by personally procuring the online tools and bill students with the cost. This is very necessary to ensure that the right online tools are used by distance learners to be able to participate in online teaching. Management could also ask the Distance Education Students Association of Ghana -DESAG to purchase the gadgets for its members. The latter option calls for the distance education institutions to make the necessary specifications available to the DESAG leadership to ensure that the right online gadgets are bought. Successful applicants could also be asked to indicate the availability or possession of online tools before admissions are granted to them.

It is recommended that special training should be provided to distance learners on how to use the online gadgets to access online content either synchronously or asynchronously. This will ensure that students will really value the importance of online gadgets for successful online delivery. The training will also allow the management to orient or sensitise students on the usefulness of online education as compared to the existing face-to-face tutorial session in vogue. The training and sensitisation programme should also capture the type of online presentation methods (synchronous and asynchronous) and how each of the methods could be navigated by distance learners. This will help reduce the level of fears or apprehension among distance learners towards the planned purely online teaching.

Management should, in the meantime, should deal with all the perceived challenges with online learning among students. After this, management can continue with the blended approach (both face-to-face and online learning) to such a time when students will be able to associate and appreciate the usefulness of online learning; then, a purely online learning approach could be introduced.

It is also recommended that the government of Ghana should expand the national coverage of electricity in the country. Distance learners are scattered across the length and breadth of the country. Online education largely depends on the availability of reliable electricity or power at homes and workplaces of learners. This will therefore afford distance learners to participate in online learning and minimise the fear of disruption. Distance education learners should also partner with the telecommunication companies in Ghana or the government of Ghana to provide a zero-rated Subscriber identification Module (SIM) card for distance learners to be able to participate freely in online learning.

## Limitations and recommendations for future studies

This study was limited to undergraduate distance students in Ghana. Therefore, future studies could consider online intention among other stakeholders such as course tutors, module writers, and administrative staff working on distance education in Ghana. Also, future studies could consider the blended approach (both face-to-face and online learning approaches) or online intention among postgraduate students in Ghana. Comparative studies among all the stakeholders also remain a plausible option to investigate further.

## Supplementary Information

**Additional file 1.** Survey instrument for the study.

## Data Availability

The datasets generated during generated and/or analyzed during the current study are available from the corresponding author on reasonable request.

## Abbreviations

AOG: Availability of gadgets
AVE: Average variance extracted
BI: Behavioural intention
CFA: Confirmatory factor analysis
DESAG: Distance Education Students Association of Ghana
FOG: Functionality of gadgets
FTF: Face to face
HTMT: Heterotrait–Monotrait ratio
OLPM: Online teaching/presentation method
PLS: Partial least squares
PLS-SEM: Partial least squares structural equation modelling
SEM: Structural equation modelling
SIM: Subscriber identification module (SIM)
TRA: Theory of reasoned action
USE: Use of online learning platform
VIF: Variance inflated factor
