# Supplementary material for: Modelling the perspectives of distance education students towards online learning during COVID-19 pandemic
Source: Smart Learn Environ. 2022 Mar 9;9(1):13. doi: 10.1186/s40561-022-00193-y (PMC8905565; doi:10.1186/s40561-022-00193-y)
Supplement: Supplementary file 1 — Additional file 1. Survey instrument for the study. [file 40561_2022_193_MOESM1_ESM.docx]

**Appendix A: Questionnaire for data collection**

**UNIVERSITY OF CAPE COAST**

**COLLEGE OF DISTANCE EDUCATION**

**ARTS AND SOCIAL SCIENCES UNIT-CoDE**

**QUESTIONNAIRE ON “COVID-19 PANDEMIC AND ONLINE EDUCATION BY THE COLLEGE OF DISTANCE EDUCATION: PROSPECTS AND CHALLENGES.”**

**QUESTIONNAIRE FOR STUDENTS AND DESAG EXECUTIVES**

Dear Respondent,

This questionnaire is intended to solicit information on **“Covid-19 pandemic and online education by the College of Distance Education: Prospects and challenges.”** It would be very much appreciated if you could provide your candid impression about each item as to how it relates to you. Every piece of information you provide will be kept strictly confidential and would be used solely for academic purposes and to help Management of CoDE in its quest to introduce online education for its distance programmes at all levels. Thank you in anticipation for your co-operation.

**PART A: DEMOGRAPHIC CHARACTERISTICS**

1. Please tick your category:

1.Student [ ] 2. DESAG Executive [ ] 3. Class Rep [ ] 4. DESAG Centre Rep [ ]

2. Gender: 1. Male [ ] 2. Female [ ]

3. Please indicate your level as a student .....................................................

4. Category of academic programme: 1. Education [ ] 2. Business [ ] 3. Maths and Science [ ]

5. Level of academic programme: 1. Diploma [ ] 2. Bachelor Degree [ ]

**PART B: AVAILABILITY AND FUNCTIONALITY OF ONLINE TOOLS/GADGETS**

6. Please tick to indicate whether you have (**availability**) the following gadgets and are functional to support an online teaching and learning.

| No | Gadget | Not available | Not functioning | Available | Functioning |
| --- | --- | --- | --- | --- | --- |
| AF1 | Android phone |  |  |  |  |
| AF2 | Computer/laptop |  |  |  |  |
| AF3 | Internet facility at home (modem, Wi-Fi or MiFi, hotspot) |  |  |  |  |
| AF4 | Internet facility at work (modem, MiFi, Wi-Fi, hotspot, etc.) |  |  |  |  |
| AF5 | Availability of electricity at home |  |  |  |  |
| AF6 | Availability of electricity at work |  |  |  |  |

**PART C: ONLINE LEARNING METHODS**

7. Please tick the online method you are able to participate in.

| **No** | **Online Method** | **Can participate** | **Cannot participate** |
| --- | --- | --- | --- |
| OLM1 | Video /Voiceover PPT (PowerPoint Slides) |  |  |
| OLM2 | Audio recorded lecture |  |  |
| OLM3 | Live online lecture through UCC e-learning platform, Zoom or Google meet. |  |  |

**PART D: PERCEIVED USEFULNESS (PROSPECTS) OF ONLINE LEARNING.**

1. Please indicate your level of agreement to the following statements on the prospects of online learning for the College of Distance Education if the College decides to go online where **SD= Strongly Disagree, D= Disagree, A= Agree, and SA =Strongly Agree.**

| **No** | **Items** | **SD** | **D** | **A** | **SA** |
| --- | --- | --- | --- | --- | --- |
| P1 | Online learning by CoDE will enable tutors to teach students better. |  |  |  |  |
| P2 | E-learning by CoDE will afford me the opportunity to have more time to interact with tutors as compared to face-to-face. |  |  |  |  |
| P3 | E-learning by CoDE will save money since there will be no need to travel to a specific geographical location to participate in class. |  |  |  |  |
| P4 | Online teaching/learning will help reduce the difficulties associated with travelling to the study centre for face-to-face teaching. |  |  |  |  |
| P5 | Online teaching will be more flexible for me than face-to-face teaching. |  |  |  |  |
| P6 | E-learning will make learning take place anytime, anywhere and greatly improve my knowledge retention. |  |  |  |  |
| P7 | E-learning will be helpful to slow learners as they can learn on their own pace to eliminate the frustrations, they have with themselves, their fellow learners, and the courses they offer. |  |  |  |  |
| P8 | E-learning will help meet the learning needs of different categories of students as compared to face-to-face. |  |  |  |  |
| P9 | Online learning will help me acquire other necessary online skills. |  |  |  |  |
| P10 | Online learning will help reduce school fees |  |  |  |  |
| P12 | E-learning is the way to go for students of CoDE now. |  |  |  |  |
| P13 | I agree with CoDE to start online teaching even if I am comfortable with the face-to-face mode. |  |  |  |  |
| P14 | I will be available and ready to be part of the e-learning if it is introduced while I am still a student. |  |  |  |  |

**PART E: CHALLENGES ASSOCIATED WITH ONLINE LEARNING**

1. Please rate the level of challenges with regards to the introduction of online teaching and learning by CoDE in the near future by ticking the appropriate column against the statement below where **SD= Strongly Disagree, D= Disagree, A= Agree, and SA =Strongly Agree.**

| **No.** | **Items** | **SD** | **D** | **A** | **SA** |
| --- | --- | --- | --- | --- | --- |
| F1 | I might not be able to prepare well for e-learning. |  |  |  |  |
| F2 | I might not be able to participate in e-learning effectively as compared to the face-to-face. |  |  |  |  |
| F3 | I might lose the opportunities to socially collaborate with other students if CoDE introduces purely online learning. |  |  |  |  |
| F4 | Keeping students interested in the course content during e-learning lessons could be challenging for students. |  |  |  |  |
| F5 | Making students feel included as a member of the class during online teaching might not be possible. |  |  |  |  |
| F6 | Students might not be motivated during online teachings as compared to face-to-face teaching. |  |  |  |  |
| F7 | Finding a quiet place to join online classes could be challenging for me. |  |  |  |  |
| F8 | Combining online learning with family responsibilities, especially if I have to join online classes from home will be very challenging for me. |  |  |  |  |
| F9 | Combining online teaching with work schedules, especially if I have to join online classes from the workplace might be very challenging for me. |  |  |  |  |
| F10 | Online classes could reduce the availability of tutors to help students. |  |  |  |  |
| F11 | I might perform poorly academically because of e-learning/online classes |  |  |  |  |
| F12 | I have not been trained for online learning. |  |  |  |  |
| F12 | I do not have the prerequisite skills for online classes. |  |  |  |  |
| F13 | I might not get the requisite logistics for the online lessons (computer, laptop, tablets and Android phone). |  |  |  |  |
| F14 | Unreliable internet connectivity could disrupt teaching and learning whiles online |  |  |  |  |
| F15 | Unreliable power-electricity could disrupt online teaching and learning. |  |  |  |  |
| F16 | Online teaching will not allow students to be able to discuss topics with their classmates. |  |  |  |  |

**PART F: ONLINE LEARNING PREFERNESS/INTENTION**

1. Please indicate your level of agreement to the following statements on areas or activities of CoDE you prefer to be moved to the online platform by ticking the appropriate column against the statement below where **SD= Strongly Disagree, D= Disagree, A= Agree, and SA =Strongly Agree.**

| No. | Items | SD | D | A | SA |
| --- | --- | --- | --- | --- | --- |
| OLP1 | I prefer that classes/teaching should is done online. |  |  |  |  |
| OLP2 | I prefer that quizzes or continuous assessment is done online. |  |  |  |  |
| OLP3 | I prefer that end of semester examination is done online. |  |  |  |  |
| OLP4 | I prefer that supervision of project work/dissertation is done online. |  |  |  |  |
| OLP5 | I prefer that some aspects of teaching practice is done online. |  |  |  |  |
| OLP6 | I prefer that some aspects of science practicals should be done online. |  |  |  |  |

11. Please rate the level of your overall fear/challenges and prospects/benefits (OUT OF 100%) for the

quest to introduce e-learning by CoDE,

| **Aspects** | **Percentage (100)** |
| --- | --- |
| Fear/challenges |  |
| Prospects/benefits |  |

**THANKS FOR YOUR VALUABLE RESPONSE AND TIME.**
